# C-953-04. Financial Ties in Burn Product Research: A Systematic and Network Analysis of Industry Influence

**DOI:** 10.1093/jbcr/irag033.128

**Published:** 2026-04-06

**Authors:** Mare G Kaulakis, Kian Daneshi, Sarah M Tepe, Hilary Y Liu, Jose Antonio Arellano, Christopher Fedor, Haig A Yenikomshian, T Justin Gillenwater, Jenny A Ziembicki, Francesco M Egro

**Affiliations:** University of Pittsburgh Medical Center, Mercy Burn Center, Pittsburgh, PA; School of Population Health and Medicine, University of Sheffield, London, England; University of Pittsburgh Medical Center, Pittsburgh, PA; University of Pittsburgh Medical Center, Mercy Burn Center, Pittsburgh, PA; University of Pittsburgh Medical Center, Pittsburgh, PA; University of Pittsburgh Medical Center, Department of Plastic Surgery, Pittsburgh, PA; Division of Plastic and Reconstructive Surgery, Keck School of Medicine, University of Southern California, Los Angeles, California; Division of Plastic and Reconstructive Surgery, Keck School of Medicine, University of Southern California, Los Angeles, California; University of Pittsburgh Medical Center, Pittsburgh, PA; University of Pittsburgh Medical Center, Pittsburgh, PA

## Abstract

**Introduction:**

Financial conflicts of interest (COI) in biomedical research have the potential to influence study outcomes and clinical recommendations. Cell-based autologous skin regeneration technology has been widely studied, yet the extent to which its authors receive industry payments remains unclear. Understanding the financial relationships between researchers and the manufacturer of autologous skin cell suspension, as well as other industry entities, is critical for assessing the scope and nature of industry involvement in this field. This study aims to systematically evaluate COI among authors publishing on the product by analyzing publicly available payment data.

**Methods:**

All authors who have published research on of autologous skin cell suspension were identified, and data were collected on publication count, year of publication, and Open Payments database presence. Payment data were collected from Open Payments for all years, including total payments received from all companies, total payments from Avita Medical, and a detailed breakdown of Avita-specific payments (e.g., consulting fees). Additionally, a co-authorship network was constructed to evaluate patterns of collaboration and author influence within autologous skin cell suspension research. This dataset was then analyzed utilizing R Studio and Stata to assess trends in industry payments.

**Results:**

We identified 193 unique authors. Of these, 103 (53.4%) formed a connected co-authorship network, indicating a core group of highly collaborative researchers. 82 authors (42.5%) had Open Payments profiles, and 43 (22.3%) received payments from Avita Medical. Only 47 authors (24.4%) disclosed conflicts of interest. The median payment from Avita was $2026 (IQR: $504–$11 941), while the mean was markedly higher at $32 798, with consulting fees totaling $342 842 since 2017. A strong association was observed between autologous skin cell suspension publication count and total Avita payments (R^2^ = 0.69, *p*<.01). Authors who were more connected in the co-authorship network (higher centrality) tended to receive more funding (ρ = 0.30, *p*=.037). However, this relationship disappeared once controlled for how many related papers each author had published, suggesting that funding is more closely tied to productivity than collaboration patterns.

**Conclusions:**

Although not all authors had Open Payments accounts, a statistically significant relationship was able to be drawn between the number of articles published and the compensation from Avita. A strong association was noted between funding and the number of publications, highlighting the importance of defining financial connections when assessing the clinical research environment.

**Applicability of Research to Practice:**

This research highlights the influence that industry has on product development and use. In an era of new technologies and product development, it is important to understand the financial ties between industry and consumer.

**Funding for the study:**

N/A.

